# Recruitment for a digitally based follow-up program for people with chronic obstructive pulmonary disease: a pilot cluster randomized controlled trial

**DOI:** 10.1186/s40814-026-01832-8

**Published:** 2026-05-09

**Authors:** Haakon Kristian Kvidaland, Marjolein Memelink Iversen, Bente Frisk, David A. Richards, Kirsten Lomborg, Christine Råheim Borge, Beate-Christin Hope Kolltveit

**Affiliations:** 1 Department of Health and Caring Sciences, Faculty of Health and Social Sciences, Western Norway University of Applied Sciences, Bergen, Norway; 2 Department of Health and Functioning, Western Norway University of Applied Sciences, Bergen, Norway; 3 Faculty of Health and Life Sciences, University of Exeter, Exeter, EX1 2LU UK; 4 Department of Clinical Medicine, University of Copenhagen, Copenhagen, Denmark; 5 Copenhagen University Hospital, Steno Diabetes Center, Copenhagen, Denmark; 6 Department of Public Health and Interdisciplinary Health Sciences, University of Oslo, Oslo, Norway; 7 Department of Patient Safety and Research, Lovisenberg Diaconal Hospital, Oslo, Norway; 8 Lovisenberg Diaconal University College, Oslo, Norway

**Keywords:** Primary healthcare, Chronic obstructive pulmonary disease, Pilot, Feasibility, Recruitment, Guided Self-Determination, Digital follow-up, Complex intervention

## Abstract

**Background:**

Recruiting participants for clinical trials in primary care settings remains challenging, not least for interventions targeting individuals with chronic obstructive pulmonary disease (COPD). Clinical outcomes and the effects of interventions receive considerable attention compared to factors influencing recruitment success. Identifying and addressing possible barriers and facilitators is essential for optimizing recruitment strategies and ensuring the feasibility of trials.

**Aim:**

This study aimed to assess uncertainties regarding recruitment for a digitally based Guided Self-Determination (GSD) follow-up program for people with COPD, prior to a fully powered cluster randomized controlled trial (cRCT), and to explore characteristics of primary care practices and patients willing to be included in the study.

**Methods:**

The intervention was a 9-month digitally based follow-up program performed with the GSD counseling method, conducted by nurses in primary care practices. Eligible primary care practices were invited to participate, and participants were recruited through their respective practices. The primary feasibility outcome was recruitment, assessed at both the practice (cluster) and patient (participants) level. Eligible participants were people between 35 and 80 years diagnosed with COPD. Baseline characteristics included demographic data, COPD Assessment Test, Dyspnea-12, WHO-5 Well-being Index, and Health Education Impact Questionnaire domains. Progression criteria *Go*, *Amend*, and *Stop* were applied to assess feasibility.

**Results:**

Recruitment occurred from May to December 2024. Of 16 eligible practices, 12 (75%) responded, of which ten (83%) agreed to participate, corresponding to 63% of the eligible practices. A total of 64 participants with COPD were recruited, 28 in the intervention and 36 in the control clusters. The overall median recruitment rate was 6.5 participants per cluster, 7 in the intervention clusters and 6 in the control clusters. One of three progression criteria, cluster recruitment, met the target Go, while cluster sizes and participant recruitment met the amended criteria.

**Conclusion:**

Recruitment was deemed feasible. However, successful cluster-level recruitment does not necessarily ensure effective patient enrollment, highlighting the necessity for amendments before a fully powered cRCT. Future research should integrate qualitative data obtained from patients and primary care practitioners to enhance the understanding of recruitment strategies.

**Trial registration:**

ClinicalTrials.gov (NCT06401512). Registered 23 April 2024, https://clinicaltrials.gov/study/NCT06401512.

**Supplementary Information:**

The online version contains supplementary material available at 10.1186/s40814-026-01832-8.

## Key messages regarding feasibility

- There is limited knowledge about the feasibility of recruiting participants for complex interventions within primary healthcare settings for people with chronic obstructive pulmonary disease.
- The recruitment has shown to be feasible, with both primary care practices and patients demonstrating a willingness to participate. However, variations in cluster sizes and inconsistent enrollment highlight the necessity for clearer recruitment targets and enhanced support at the cluster level.
- To ensure consistent patient inclusion across all sites, the fully powered cluster randomized controlled trial should implement structured recruitment protocols, establish clear enrollment targets for each cluster, and allocate dedicated resources to support the clusters.

## Background

Chronic obstructive pulmonary disease (COPD), characterized by non-reversible airflow obstruction with dyspnea, cough with sputum production, and fatigue as the main symptoms, remains a leading cause of mortality and morbidity worldwide [1–4]. In adults aged ≥40 years, the prevalence of the diagnosis is estimated at roughly 10% globally [2] and 6 to 7% in Norway [5]. The disease often affects people’s ability to undertake daily activities and may require significant lifestyle changes to manage the challenges of living with the disease [6]. Regular follow-up, emphasizing person-centered care through self-management support and counseling, can reduce symptoms and exacerbations due to COPD [7, 8]. However, despite established guidelines, follow-up for individuals with COPD is frequently suboptimal globally [9], and attendance at regular follow-up visits is low, also in Scandinavian countries [10, 11]. Furthermore, in Norway, a strong association between outpatient contacts and subsequent hospital admissions suggests that patients primarily seek primary care when their condition changes or worsens, rather than on a regular basis [12]. Such suboptimal care, marked by missed or inadequate follow-up visits, places patients at an increased risk of complications and other adverse events [13–15]. As the population gradually ages [16], it is likely that the prevalence of COPD, along with other chronic conditions, will increase both in Norway and globally, presenting ongoing challenges in ensuring appropriate follow-up care. Addressing these issues will require the development of more efficient follow-up procedures.

Guided Self-Determination (GSD) is a person-centered theory-driven counseling program founded on synthesizing self-determination, life skills, and humanistic values theory [17]. Hence, GSD intends to empower people with chronic conditions to self-manage their health by enhancing self-determination and life skills [18]. Results from an integrated review assessing this method have demonstrated improvements in perceived competence, autonomous motivation, psychosocial well-being, decreased distress, and increased motivation among people with diabetes, schizophrenia, cancer, chronic pain, and survivors of invasive care [19]. A qualitative study has found indications that the GSD method is also useful among people with COPD [20]. However, the results from this study showed that it was challenging for healthcare professionals to implement the GSD intervention due to the increased paperwork and logistics required for follow-up [20]. These findings suggest the need for further investigation and the potential amendment of a GSD follow-up program for people with COPD. A digitally based GSD follow-up program may ease the workload for healthcare professionals while harnessing the possible benefits of using the GSD method in follow-up consultations.

### Knowledge gap and uncertainties

Despite the emphasis on self-management in COPD-related follow-ups, no randomized controlled trials have been conducted using the GSD method in primary healthcare for this patient population. Consequently, uncertainties remain about the willingness of both primary care practices and patients to participate in such a trial, as well as the most effective strategies for recruitment. More broadly, a significant knowledge gap exists regarding effective recruitment strategies in primary healthcare, as clinical trials tend to focus on outcomes and intervention effects, while the recruitment process receives limited attention [21]. Therefore, uncertainty related to recruitment must be addressed before potentially progressing with a fully powered clinical trial. This is particularly important when evaluating the GSD method for people with COPD in a primary healthcare context.

### Study aim

This study aimed to assess uncertainties regarding recruitment for a digitally based GSD follow-up program for people with COPD, prior to a fully powered cluster randomized controlled trial (cRCT), and to explore characteristics of primary care practices and patients willing to be included in the study.

### Research questions

What proportion of approached and invited primary care practices will likely agree to participate in a clinical trial using the GSD method in follow-ups for patients with COPD?

What is the likely cluster size, i.e., participant recruitment rate per practice?

What are the characteristics of primary care practices willing to participate in a digitally based follow-up program for people with COPD?

What proportion of approached and invited people with COPD will likely agree to participate in a digitally based follow-up program with their primary care practice?

What are the characteristics of people with COPD who are willing to participate in a digitally based follow-up program with their primary care practice?

## Methods

### Study design

This was a pilot cRCT, conducted in primary healthcare practices in western Norway for people with COPD, to assess the feasibility and acceptability of recruitment procedures for a later fully powered cRCT. The modified Medical Research Council’s (MRC) framework for complex interventions was followed, focusing on the initial phases of intervention development and piloting [22, 23]. The framework emphasizes exploring key aspects of uncertainty and acceptability, which is crucial in these early phases. Progression criteria were applied to assess the feasibility of recruitment.

### Progression criteria

The progression criteria are based on the traffic light system described by Avery et al. [24], which uses three categories: *Go* (green), *Amend* (amber), and *Stop* (red). The predefined criteria are inspired by previous research [25, 26] and the work by Borrelli [27]. A *Go* decision indicates that the study should proceed, with minor amendments if necessary. An *Amend* decision requires further discussions within the project group to determine whether modifications can be implemented to enable progression. A *Stop* decision suggests that proceeding with a fully powered trial is not advisable unless clear contextual modifications or design changes are identified and addressed. The three progression criteria for this study are presented in Table 1.

**Table 1:** Progression criteria for the study

Concept	Data collection	Relevant sources	Progression criteria		
			Stop	Amend	Go
Cluster recruitment	Data obtained by project group	[28, 29]	If ≤25% of invited practices agree to participate	If 25–50% of invited practices agree to participate	If ≥50% of invited practices agree to participate
Cluster size	Data obtained by project group	[28, 30, 31]	If the median cluster size is ≤4 participants	If the median cluster size is 4–7 participants	If the median cluster size is ≥8 participants
Participant recruitment	Recruitment data reported by the clusters	[30–32]	If ≤25% of invited patients agree to participate	If 25–50% of invited patients agree to participate	If ≥50% of invited patients agree to participate

The progression criteria are based on the traffic light system described by Avery et al. [24], and predefined criteria are inspired by previous research [25, 26] and the work by Borrelli [27]

### Sample size

It is not appropriate to undertake formal sample size calculations for feasibility studies where the intention is not to undertake between-group inferential statistical analyses [33]; however, a sample size of approximately 60 participants, with 30 in each arm, is regarded as suitable for testing procedural uncertainties such as recruitment [34, 35]. A sample size of 75 was targeted to accommodate attrition of 25%, with approximately 37 in each arm [36–38]. With a targeted recruitment rate of eight participants per cluster, ten clusters were regarded as necessary to achieve the target of 75 participants recruited.

### Inclusion criteria

#### Clusters

The cluster units were primary care practices. Practices with two or more physicians, at least one nurse, and a minimum of ten patients diagnosed with COPD were eligible for inclusion.

#### Participants

Eligible participants in the study were patients between 35 and 80 years of age who had previously been diagnosed with COPD and were followed up at an included primary care practice. The age range captures the typical onset of COPD in midlife, while also including older adults, who represent the majority of cases, ensuring comprehensive representation [39]. Additionally, participants needed to have their own personal BankID, a digital personal identification tool similar to systems like Digital Signature or Electronic ID.

### Exclusion criteria

Exclusion criteria for participants were other severe somatic diseases (i.e., palliative care, heart failure where physical activity is not recommended, end-stage renal disease) and severe psychiatric diagnoses (e.g., severe depression, bipolar disorder, schizophrenia) preventing engagement in the intervention. In addition, participants who could not provide informed consent, which entails a full understanding of the study details, and/or did not have the ability to write, speak, and understand Norwegian were excluded.

### Recruitment

The recruitment period was between May and December 2024. Recruitment was done by contacting leaders of one or more primary care practices in municipalities. Municipalities known or presumed to have nurses employed at the practices were contacted. If found eligible, they received an approximately 3-min animated introduction video, along with a corresponding brochure about the project, prior to a meeting and a request for inclusion. Physicians and nurses at the included clusters identified and established contact with eligible patients. Patients received information about the project and gave their consent digitally using their BankID. Each practice designated a contact person for communication, who received information at each step of the process. No economic incentives were provided for either clusters or participants.

### Randomization, allocation concealment, and blinding

The clusters were randomized to either the intervention (digital GSD follow-up program plus treatment as usual) or the control group (treatment as usual alone). Clusters were randomized in a parallel design with a 1:1 allocation ratio, using block randomization with a block size of two to ensure balanced group sizes throughout the trial. For randomization across municipalities, pairs were stratified into groups based on the municipality’s population size: small (≤3000 inhabitants), medium (3000–10,000 inhabitants), and large (≥10,000 inhabitants). Each cluster was randomly assigned a number, and randomization was performed using the statistical software Stata, led by a statistician blinded to the characteristics of the clusters. As an intervention in primary healthcare, neither the clusters nor the participants were blinded to the intervention.

### Intervention—the digital GSD follow-up program

**Fig. 1:**
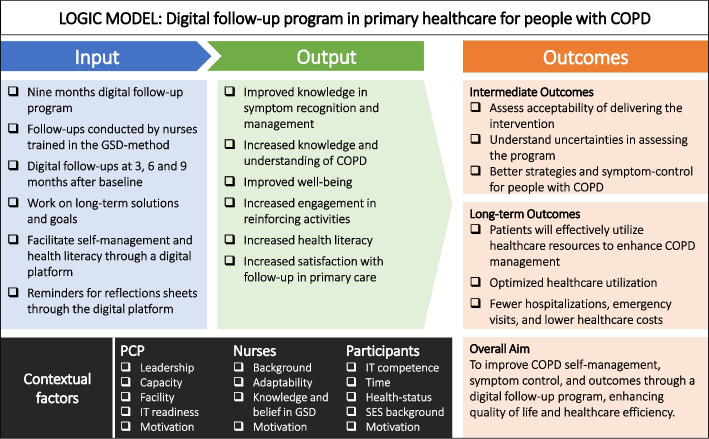
Logic model for the digitally based Guided Self-Determination (GSD) follow-up program for people with chronic obstructive pulmonary disease (COPD). Abbreviations: COPD: chronic obstructive pulmonary disease; GSD: Guided Self-Determination; PCP: primary care practices; SES: socioeconomic status

The digital GSD follow-up program consisted of four scheduled consultations with a nurse. The initial consultation was an on-site annual check-up at baseline, followed by three digital follow-up consultations at 3, 6, and 9 months (see Additional file 1). Consultations were conducted using a digital platform, facilitated by reflection sheets that stimulated written reflection in the context of GSD. Before and between consultations, participants were given access to reflection sheets on the digital platform, which were distributed across four steps, each focusing on a specific area (see Additional file 2). In addition to the reflection sheets, participants received tailored information specifically designed for people with COPD, developed in collaboration with a user organization and representatives with COPD. This information, including both video and written content, was delivered through the digital platform, covering topics such as medication use, flu vaccination, physical activity, nutrition, and breathing techniques, all in adherence to established guidelines. Nurses were encouraged to monitor their patient’s progress, utilizing this data to facilitate person-centered and meaningful consultations. The logic model for the intervention is depicted in Fig. 1, outlining core input, mechanisms of change, output, and anticipated outcomes to inform the design and evaluation of the intervention.

### Treatment as usual

Treatment as usual refers to the standard care routinely provided to patients without imposing restrictions on treatment options. This included routine check-up appointments as scheduled with their respective primary care practice. Participants in a control cluster received only treatment as usual, ensuring they continued their typical care regimen without additional interventions.

### Outcomes

The outcomes of this study were the cluster recruitment rate, cluster sizes, and participant recruitment rate.

### Measurement for cluster and participant characteristics

#### Clusters

To describe the baseline characteristics of the included clusters, data were collected on the type of municipality in which each practice was located (e.g., urban, rural, or small town), as well as the number of patients, nurses, and physicians at each practice.

#### Participants

Baseline data were collected from participants, including demographic characteristics and patient-reported outcome measures (PROMs). These outcomes were measured by COPD Assessment Test (CAT), the World Health Organization Well-Being Index (WHO-5), Dyspnea-12 questionnaire (D-12), and the Health Education Impact Questionnaire (HeiQ).

Patient-reported outcomes

CAT is an 8-item, disease-specific, reliable, and validated questionnaire that assesses the impact of COPD on a patient’s health [40]. Each item is scored from 0 to 5, with a total score ranging from 0 to 40. Higher scores indicate a greater impact on health status, and a minimum clinically important difference (MCID) for the CAT score is two points [41, 42].

WHO-5 is a brief, valid, and reliable self-reported questionnaire used to assess well-being [43–45]. It consists of five items and each item is scored on a scale from 0 (none of the time) to 5 (all of the time), resulting in a total score ranging from 0 to 25. The score is multiplied by 4 to find a range from 0 to 100, whereas a score of 0 being the lowest well-being score and 100 being the highest, with an MCID of 10% [43].

D-12 is a validated and reliable questionnaire to measure the severity of dyspnea experienced by people with COPD [46]. It consists of 12 items that capture both physical and affective aspects of breathlessness. Each item is scored from 0 (no breathlessness) to 3 (severe breathlessness), resulting in a total score ranging from 0 to 36, with higher scores indicating greater severity of dyspnea [47]. There is no reported MCID for D-12.

HeiQ is a validated tool designed to assess the outcomes of health education and self-management programs [48]. It comprises 40 items divided into eight domains that measure different aspects of health education impact, including health-directed activities, positive and active engagement in life, emotional well-being, self-monitoring and insight, constructive attitudes and approaches, skill and technique acquisition, social integration and support, and health service navigation. Each item is scored from 0 (strongly disagree) to 4 (strongly agree), and a higher score indicates better outcomes [49]. There is no MCID for HeiQ; however, a group-change effect size of 0.2–0.5, 0.5–0.8, and higher than 0.8 is conventionally considered small, medium, and large, respectively [50].

### Statistical analyses

All analyses were performed using R 4.4.3. Recruitment of practices was reported in terms of the number and proportion of primary care practices approached, the number of practices that responded, and the number that accepted to participate. Recruitment of participants was reported in terms of the number of participants screened, found eligible, contacted, and who provided written consent. Descriptive statistics were used to summarize results and characteristics at both the cluster and participant levels. Continuous variables were reported as means and standard deviations (SD) or medians with interquartile ranges (IQR) or ranges. Categorical variables were summarized using counts and percentages. All data were assessed at baseline.

### Ethical considerations

The project was carried out in accordance with the Declaration of Helsinki. Ethical approval was obtained from the Western Norway Regional Committees for Medical and Health Research Ethics (REK 2023/656382) and the Norwegian Agency for Shared Services in Education and Research (281272). The trial is registered at ClinicalTrials.gov (Study ID: NCT06401512). All collected data are securely stored on the Western Norway University of Applied Sciences research server. All participants provided written, informed consent before participation, and all procedures adhered strictly to relevant guidelines and regulations. Data are reported in accordance with the Consolidated Standards of Reporting Trials (CONSORT) extension for pilot and feasibility trials [34].

## Results

### Recruitment

**Fig. 2:**
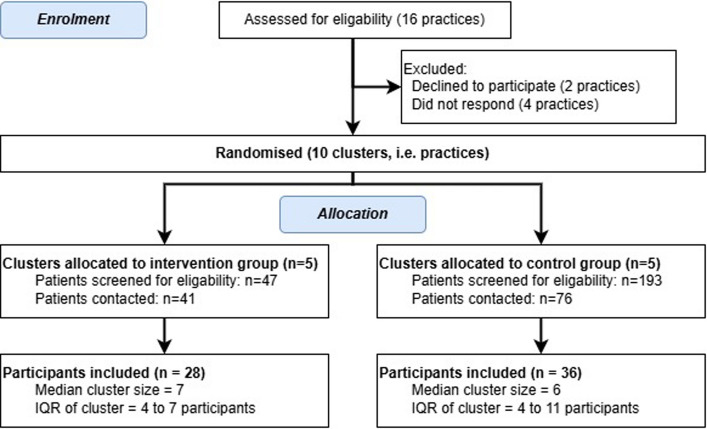
CONSORT flow diagram. Abbreviation: IQR: interquartile range

A detailed description of the recruitment process is presented in Fig. 2. Summarized, 16 primary care practices were identified as eligible. Of these, 10 (62.5%) agreed to participate, two (12.5%) declined, and four (25.0%) did not respond. The reasons given for declining were lack of capacity at one practice, and a perceived lack of necessity to change current routines at the other.

In total, 64 patients were included, 28 (44%) in the intervention group and 36 (56%) in the control group. Ten primary care practices participated, five in each group. The overall median cluster (practice) size was 6.5 participants (range: 2–13). Respectively, the median cluster size was 7 patients in the intervention clusters (range: 2–8) and 6 patients in the control clusters (range: 4–13). Two clusters in each group, a total of 4 out of 10, recruited fewer than five participants.

The recruitment rate among the invited participants was 68% in the intervention group and 47% in the control group, with an overall recruitment rate of 55%. No explicit reasons were provided by those who did not respond. However, participants who declined to participate described barriers such as an overwhelming life situation, other treatments plans, lack of BankID or digital literacy, and reluctance to engage with anything related to the disease.

### Characteristics

#### Clusters

The clusters had an overall median size of approximately 4350 total registered patients (range: 1150–7250) and six physicians (range: 3–10). Of the ten included practices, six were located in rural municipalities, two in small towns, and two in urban areas. See Table 2 for a detailed overview of clusters characteristics.

**Table 2:** Characteristics for clusters in the pilot cluster randomized controlled trial

	Intervention	Control	Total
Number of clusters, *n* (%)	5 (50)	5 (50)	10
Number of staff, median (range)			
Physicians	7 (3 to 9)	6 (3 to 10)	6 (3 to 10)
Nurses	3 (1 to 18)	2 (2 to 4)	2.5 (1 to 18)
Other	4 (1 to 6)	4 (0 to 8)	4 (0 to 8)
Patient list size, median (range)	4745 (1200 to 7260)	3950 (1150 to 6500)	4348 (1150 to 7260)
Practice location, *n* (%)			
Rural municipality	3 (60)	3 (60)	6 (60)
Small town	1 (20)	1 (20)	2 (20)
Urban area	1 (20)	1 (20)	2 (20)

#### Participants

The demographic characteristics of the included participants are presented in Table 3.

**Table 3:** Characteristics for participants at baseline

	Intervention *n* = 28	Control *n* = 36	Total *n* = 64
Participants, *n* (%)			
Total	28 (44)	36 (56)	64
Female	15 (54)	18 (50)	33 (52)
Male	13 (46)	18 (50)	31 (48)
Age (years), mean (SD)			
Total	65.7 (8.3)	69.3 (6.7)	67.7 (7.6)
Female	68.0 (7.3)	68.0 (6.3)	68.0 (6.7)
Male	63.0 (8.8)	70.6 (6.9)	67.4 (8.5)
BMI (cm)^a^, mean (SD)			
Total	26.3 (5.4)	27.5 (4.6)	27.0 (5.0)
Female	25.1 (4.8)	28.5 (4.7)	26.9 (5.0)
Male	27.7 (5.9)	26.9 (5.0)	27.0 (5.1)
Education^b^, *n* (%)			
Lower secondary	10 (36)	10 (29)	20 (32)
Upper secondary	14 (50)	19 (54)	33 (52)
Higher secondary	4 (14)	6 (17)	10 (16)
Employment status^b^, *n* (%)			
Fulltime employed	5 (18)	6 (17)	11 (17)
Parttime employed	2 (7)	2 (6)	4 (6)
Disability benefit	7 (25)	6 (17)	13 (21)
Pensioner	14 (50)	21 (60)	35 (56)
Marital status^b^, *n* (%)			
Married	10 (36)	21 (60)	31 (49)
Partner	5 (18)	5 (14)	10 (16)
Divorced/separated	7 (25)	3 (9)	10 (16)
Single	4 (14)	2 (6)	6 (9.5)
Widow/widower	2 (7)	4 (11)	6 (9.5)
Current living arrangement^b^, *n* (%)			
Alone	13 (46)	10 (29)	23 (37)
Not alone	15 (54)	25 (71)	40 (63)
Smoking habits^b^, *n* (%)			
Never smokers	2 (7)	2 (6)	4 (6)
Ex-smokers	16 (57)	25 (71)	41 (65)
Current smokers	10 (36)	8 (23)	18 (29)

*Abbreviation*: *SD* Standard deviation, *n* numbers

^a^Missing data: control *n* = 2

^b^Missing data: control *n* = 1

#### PROMs

All participants completed the CAT questionnaire, while 3 (5%) did not complete WHO-5 and HeiQ, and 2 (3%) did not complete D-12. Results from the included PROMs are presented in Table 4.

**Table 4:** Patient-reported outcome measures at baseline

	Intervention (*n* = 28)	Control (*n* = 36)	Total (*n* = 64)	
	Mean (SD)	Mean (SD)	Mean (SD)	Range
CAT score	18.1 (8.4)	14.7 (6.7)	16.2 (7.6)	2 to 34
WHO-5^a^	16.2 (5.1)	17.1 (4.0)	16.7 (4.5)	4 to 25
D-12^b^				
Total	10.1 (7.8)	8.3 (7.1)	9.1 (7.4)	0 to 26
Physical domain	5.7 (5.0)	4.9 (4.2)	5.3 (4.5)	0 to 18
Affective domain	4.5 (3.5)	3.4 (3.5)	3.9 (3.5)	0 to 11
HeiQ^a^				
HDA	3.0 (0.5)	3.0 (0.7)	3.0 (0.7)	1.3 to 4.0
PAEL	3.1 (0.6)	3.2 (0.6)	3.1 (0.6)	1.8 to 4.0
SMI	3.1 (0.5)	3.0 (0.4)	3.1 (0.4)	2.0 to 4.0
CAA	3.3 (0.5)	3.1 (0.5)	3.2 (0.5)	2.0 to 4.0
STA	2.9 (0.6)	3.0 (0.5)	3.0 (0.5)	2.0 to 4.0
SIS	3.1 (0.5)	3.0 (0.6)	3.0 (0.5)	1.4 to 4.0
HSN	3.1 (0.5)	3.0 (0.4)	3.0 (0.5)	2.0 to 3.8
ED	2.1 (0.6)	1.9 (0.7)	2.0 (0.7)	1.0 to 3.3

1: missing data; intervention *n* = 1, control *n* = 2. 2: missing data; intervention *n* = 1, control *n* = 1

*Abbreviations:* *SD* standard deviation, *CAT* COPD assessment test, *WHO-5* WHO Well-being Index 5, *D-12* Dyspnea-12, *HeiQ* health education impact questionnaire, *HDA* Health-directed activities, *PAEL* Positive and active engagement in life, *SMI* Self-monitoring and insight, *CAA* Constructive attitudes and approaches, *STA* Skill and technique acquisition, *SIS* Social integration and support, *HSN* Health servicesnavigation, *ED* Emotional distress

### Progression criteria evaluation

Of the three progression criteria, one (cluster recruitment) met the *Go* target, while the other two (cluster size and participant recruitment) met the *Amend* target (Table 5).

**Table 5:** Progression criteria final decision

Concept	Final decision
Cluster recruitment	Go: Out of the 16 primary care practices approached, ten (63%) agreed to participate
Cluster size	Amend: Overall, the median cluster size was 6.5, with medians of 7 and 6 in the intervention and control groups, respectively
Participant recruitment	Amend: Overall, 55% of those invited agreed to participate, with 68% in the intervention clusters and 47% in the control clusters

## Discussion

This study recruited 10 clusters and 64 participants with COPD over a 9-month recruitment period. Cluster recruitment achieved the *Go* target as more than half of the approached practices agreed to participate. Overall, participant recruitment also reached the *Go* target, but recruitment in the control group was below 50%, categorizing it in the *Amend* category. Cluster sizes were similarly categorized within the *Amend* category, with a median cluster size below the Go target. This shortfall in cluster size was the primary reason for not achieving the targeted sample size, highlighting the need for some adjustments before launching a fully powered trial.

### Progression criteria

Regarding progression criteria, the practices’ willingness to participate is deemed high. Previous research has identified several reasons for primary care practices’ lack of involvement in trials, such as excessive documentation, enrollment requirements, limited resources, and interference in practice routines [51–53]. Projects have also been terminated altogether or prematurely due to lack of involvement [54, 55]. In this study, similar challenges were identified, including recruiting fewer participants than targeted, particularly in the intervention group (criterion two), and a recruitment rate below 50% in the control group (criterion three). Despite these issues, our results suggest that a sufficient number of practices would likely be willing to participate in a future fully powered trial.

### Recruitment of practices

Three plausible reasons for the satisfying recruitment rate of clusters in this study have been identified and will be discussed. First, municipal and primary care practice leaders are incentivized by government policies to engage in research focused on digitalization, particularly following the COVID-19 pandemic [56]. As a result, leaders are encouraged to explore and implement technological advancements. The second part focuses on recognizing and considering existing barriers and facilitators in the planning and implementation of recruitment. Known barriers include time constraints, insufficient COPD follow-up routines, reluctance towards digitalization, lack of digital literacy, lack of knowledge about the trial, and role ambiguity within the practice [57–63]. A key facilitator is clinical relevance, as clinicians seem more motivated when research can help improve clinical practice, enabling them to better support their patients, enhance collaboration within the practice, and increase competency in managing the disease [57, 63, 64]. Third, in line with Moffat et al. [21], this study placed greater emphasis on recruiting practices and primary care practitioners, rather than focusing predominantly on patient recruitment. By prioritizing early engagement with practice leaders and clinicians in the recruitment process, this approach facilitated organizational commitment and willingness to participate, and thereby enhanced cluster enrollment.

However, although the progression criterion for cluster recruitment was met, it did not guarantee success in recruiting participants, highlighting an important feasibility consideration for future trials. Notably, four out of ten clusters included fewer than five participants. Since the recruitment rates were close to or higher than 50% for participants within the clusters, it seems that fewer patients were approached than expected. Hence, sufficient cluster-level follow-up is necessary to ensure enough patients are invited. Future trials should incorporate structured and targeted follow-ups on the cluster level to ensure enough patients are invited.

### Recruitment of participants

Similar to the practices, patients seemed motivated and willing to participate in a digitally based GSD follow-up program, whereas more than 60% in the intervention group and just below half in the control group agreed to participate. The discrepancy in recruitment rates and the number of patients reported screened, differing by close to 150 patients, may indicate a selection bias, whereas the clusters in the intervention group might have invited patients they perceived as most likely to agree to participate. However, the baseline characteristics of both groups align with similar trials [36, 37, 65]. It is possible that participants in an intervention group are more willing to participate in a clinical trial because they anticipate immediate benefits. Less interest among participants in the control group aligns with previous research, which finds that potential participants may decline inclusion due to awareness of potentially being allocated to a control group [66]. However, in this study, the shortfall in cluster sizes is observed in both intervention and control clusters, highlighting the need to either include more clusters or make other adjustments.

Strategies to address small cluster sizes may include implementing stricter inclusion criteria, excluding smaller primary care practices, or promoting a more extensive recruitment approach at the cluster level. Regarding digital literacy for the latter point, co-creators involved in developing an eHealth tool for people with COPD were themselves surprised at how well they managed the digital technology [67]. However, in a scoping review, lack of digital literacy was the most frequently mentioned barrier, highlighting the importance of user-friendly platforms and sufficient training [68]. Nonetheless, it is crucial that preconceived assumptions do not influence the identification or outreach to eligible patients. This is important not only to avoid excluding patients who are both willing and able to participate, but also to ensure that refinements to the intervention are informed by insights from the broader population. Amendments to improve cluster sizes could also include increasing external support, such as providing economic incentives and technical or logistical assistance during the initial phases. Providing further training to enhance recruitment capacity within each practice may also be beneficial. To better understand potential barriers and necessary adjustments, interviews with primary care practitioners and patients included in the recruitment process are needed.

The bottleneck for meeting the targeted number of participants from each practice seems to be lack of patients contacted and invited rather than low acceptance. With an overall recruitment rate close to 50%, approximately 16 participants should have been invited per cluster, whereas the average was closer to 12. One possible explanation for the lack of involvement, especially for the four clusters with low enrollment, might be that leadership decisions were made without sufficient engagement and motivation from the primary care practitioners. Loskutova et al. [61] emphasize that decisions are often taken on behalf of the practices, indicating insufficient involvement to motivate practitioners. In the control group, practitioners also received no immediate benefits, such as training in the GSD method, which may have further affected their engagement. Thus, future trials may benefit from proactive strategies to sustain cluster engagement and optimize patient enrollment, including automated reminders, designated recruitment leaders, and training programs. In retrospect, we may have focused too strongly on cluster recruitment, thereby diverting attention from patient-level engagement.

## Strengths and limitations

This study followed the modified MRC framework for complex interventions [23]. Predefined progression criteria were established to objectively evaluate the feasibility of the recruitment phase. Furthermore, because no financial incentives were offered, the willingness to participate was likely based on the study’s perceived relevance and potential benefits for both practices and patients. Including both small and large clusters in various settings enriched our insights into recruitment feasibility in diverse primary healthcare settings. However, the specific reasons why some practices recruited fewer participants remain unclear, as there was no systematic tracking of barriers, making it difficult to determine whether factors such as time constraints, competing clinical responsibilities, or lack of confidence in the digital intervention played a role. Because subgroups with language barriers or other severe diseases were excluded, selection bias may have been introduced, limiting our findings’ broader generalizability. Nonetheless, this is less of a concern in a pilot cRCT, where the primary aim is to establish feasibility and refine study procedures. However, such considerations should be considered if a fully powered trial is subsequently undertaken.

## Conclusion

This study aimed to investigate the feasibility of recruiting primary care practices and people with COPD for a digitally based GSD follow-up program, led by nurses in primary healthcare. Although 63% of approached practices agreed to participate and more than half of the invited patients were recruited, indicating a sufficient willingness to participate in such a trial, cluster sizes fell short of the target. This was primarily due to fewer patients being invited than planned. These findings highlight the need to refine recruitment procedures to enhance primary care practitioners’ engagement and motivation and ensure sufficient support throughout the recruitment process. Strategies may include adopting a more extensive recruitment approach and providing closer follow-up through reminders and increased external support. Future studies should integrate qualitative data collection at both cluster- and participant-level to better understand the recruitment process.

## Supplementary Information

Supplementary Material 1.

Supplementary Material 2.

Supplementary Material 3.

## Data Availability

In accordance with the approvals granted for this study by the Regional Committee on Medical Research Ethics and the Norwegian Data Inspectorate, the data files will be stored securely and in accordance with the Norwegian Law of Privacy Protection. A subset of the data file with anonymized data will be made available to interested researchers upon reasonable request to Beate-Christin Hope Kolltveit: beate-christin.hope.kolltveit@hvl.no.

## Abbreviations

CAT: COPD Assessment Test
CONSORT: The Consolidated Standards of Reporting Trials
COPD: Chronic obstructive pulmonary disease
cRCT: Cluster randomized controlled trial
D-12: Dyspnea-12 questionnaire
GSD: Guided Self-Determination
HeiQ: The Health Education Impact Questionnaire
IQR: Interquartile range
MCID: Minimum clinically important difference
MRC: The Medical Research Council
PROM: Patient-reported outcome measures
SD: Standard deviation
WHO-5: The World Health Organization Well-Being Index
