# Supplementary material for: Recruitment for a digitally based follow-up program for people with chronic obstructive pulmonary disease: a pilot cluster randomized controlled trial
Source: Pilot Feasibility Stud. 2026 May 9;12:91. doi: 10.1186/s40814-026-01832-8 (PMC13326346; doi:10.1186/s40814-026-01832-8)
Supplement: Supplementary file 1 — Supplementary Material 1. [file 40814_2026_1832_MOESM1_ESM.docx]

Additional file 1

Supplementary table 1: Timeline intervention and control group

| Intervention group | |
| --- | --- |
| Baseline | Information about the study and written consent.  Information about GSD as a method.  Face to face consultation in the primary care practice with an individual perspective on living with COPD.  Training in using the digital platform.  Participants will fill out questionnaires by using YouWell.  Participants will perform physical tests and measurements  Participants’ COPD-related medical information last 12 month  Consultation GSD 1: see supplementary table 2 |
| 3-month | Digital consultation with an individual perspective on living with COPD.  Consultation GSD 2: see supplementary table 2  Participants will fill out questionnaires by using YouWell.  Participants’ COPD-related medical information last 12 month |
| 6-month | Digital consultation with an individual perspective on living with COPD.  Consultation GSD 3: see supplementary table 2  Participants will fill out questionnaires by using YouWell.  Participants will perform physical tests and measurements  Participants’ COPD-related medical information last 12 month |
| 9-month | Digital consultation with an individual perspective on living with COPD.  Consultation GSD 4: see supplementary table 2  Participants will fill out questionnaires by using YouWell.  Participants’ COPD-related medical information last 12 month |
| 12-month | Participants will fill out questionnaires by using YouWell.  Participants will perform physical tests and measurements  Participants’ COPD-related medical information last 12 month |
| Control group | |
| Baseline | Information about the study and written consent.  Training in using the digital platform.  Participants will fill out the written consent by using YouWell.  Participants will fill out questionnaires by using YouWell.  Participants will perform physical tests and measurements  Participants’ COPD-related medical information last 12 month |
| 3-month | Participants will fill out questionnaires by using YouWell.  Participants’ COPD-related medical information last 12 month |
| 6-month | Participants will fill out questionnaires by using YouWell.  Participants will perform physical tests and measurements  Participants’ COPD-related medical information last 12 month |
| 9-month | Participants will fill out questionnaires by using YouWell.  Participants will perform physical tests and measurements  Participants’ COPD-related medical information last 12 month |
| 12-month | Participants will fill out questionnaires by using YouWell.  Participants will perform physical tests and measurements  Participants’ COPD-related medical information last 12 month |
