# Supplementary material for: Recruitment for a digitally based follow-up program for people with chronic obstructive pulmonary disease: a pilot cluster randomized controlled trial
Source: Pilot Feasibility Stud. 2026 May 9;12:91. doi: 10.1186/s40814-026-01832-8 (PMC13326346; doi:10.1186/s40814-026-01832-8)
Supplement: Supplementary file 2 — Supplementary Material 2. [file 40814_2026_1832_MOESM2_ESM.docx]

Additional file 2

Supplementary table 2. Overview of themes and areas to focus on in GSD reflection sheets.

| **Step 1-4** | **Focus area** |
| --- | --- |
| Step 1: Your life with the condition | Invitation to collaborate with healthcare professionals Identify key areas and priorities to focus on for your health Establish what is important for you in the short and long term “Face-to-face” consultations to establish the patient-provider relationship and finalize focus areas for the next step |
| Step 2: Further exploring your health condition and lifestyle | Receive and respond to questions sent digitally throughout step 2: Who can help you? What have you done until now? What have you tried to do? Who have you asked for help? Get access to modules in the digital platform with information and tasks to get started with various lifestyle changes (e.g. physical activity, diet) and COPD-related information (e.g. vaccinations, exacerbations, breathing advice) Digital follow-up with the nurse three months after the annual check-up. |
| Step 3: Focus on long-term actions, strategies, and solutions | Receive and respond to questions sent digitally throughout step 3 Room for the condition in your life.  Focus on how to implement suggested actions and strategies. Refining your health, focus on empowerment and goals. Digital follow-up with the nurse six months after the annual check-up. |
| Step 4: Focus on long-term actions, strategies, and solutions | Receive and respond to questions sent digitally throughout step 4 Evaluate and adjust your health strategies. Your thoughts, feelings, actions, and experiences.  Establish strategies and plans. Digital follow-up with the nurse nine months after the annual check-up. |
